# Preprocedural cerebral small vessel disease burden predicts one-year cognitive decline in patients with atrial fibrillation undergoing left atrial appendage occlusion: a prediction model and mediation analysis

**DOI:** 10.3389/fcvm.2026.1948991

**Published:** 2026-10-30

**Authors:** Zhe Wang, Liang Chen

**Affiliations:** Department of Cardiology, Nantong First People’s Hospital, Southeast University, Nantong, China

**Keywords:** atrial fibrillation, cerebral small vessel disease, cognitive decline, left atrial appendage occlusion, mediation analysis, prediction model, silent cerebral embolism

## Abstract

**Background:**

Cognitive outcomes after left atrial appendage occlusion (LAAO) are variable, and preprocedural markers of cerebral vulnerability are needed.

**Objectives:**

This study evaluated whether preprocedural total cerebral small vessel disease (CSVD) burden predicts 1-year cognitive decline after LAAO and whether silent cerebral embolism (SCE) mediates this association.

**Methods:**

This prospective, 2-center observational cohort study enrolled consecutive patients with atrial fibrillation (AF) scheduled for LAAO at the First Affiliated Hospital of Nanjing Medical University and Nantong First People's Hospital between January 2023 and June 2025. Total CSVD burden score was calculated from preprocedural brain magnetic resonance imaging (MRI). Cognitive function was assessed using the Montreal Cognitive Assessment (MoCA) and Mini-Mental State Examination (MMSE) before the procedure and at 1 year. The primary outcome was cognitive decline, defined as a decrease of at least 2 points in MoCA. SCE was assessed on follow-up MRI. Multivariable logistic regression, prediction modeling, bootstrap internal validation, and mediation analysis were performed.

**Results:**

Among 110 patients, the mean age was 69.10 ± 6.48 years, 64.5% were men, and the median total CSVD burden score was 1.50, interquartile range 1.00 to 2.00. During 1-year follow-up, SCE was detected in 42 patients, 38.2%, and cognitive decline occurred in 32 patients, 29.1%. In a parsimonious multivariable model adjusted for age, sex, hypertension, and baseline MoCA score, total CSVD burden score was independently associated with MoCA-defined cognitive decline, odds ratio: 5.39 per 1-point increase; 95% CI: 2.91 to 11.75; *P* < 0.001. Adding total CSVD burden score to the clinical model significantly improved predictive performance, increasing the apparent area under the curve (AUC) from 0.670 to 0.890 and the optimism-corrected AUC from 0.608 to 0.863. Exploratory mediation analysis suggested a partial mediating role of SCE, with an estimated proportion mediated of 28.3%. MMSE-based sensitivity analyses were broadly consistent.

**Conclusions:**

Preprocedural total CSVD burden score was associated with 1-year cognitive decline after LAAO and improved prognostic discrimination beyond clinical variables. SCE may partly mediate this association, supporting total CSVD burden score as a potential marker of vulnerability to postprocedural cognitive decline.

## Introduction

Atrial fibrillation (AF) and cognitive impairment are common in aging populations and share multiple vascular risk factors (1). Observational studies have consistently linked AF to an increased risk of cognitive decline and dementia (2–4). Although overt stroke is an important mechanism linking AF to cognitive impairment, this association persists in patients without clinically recognized stroke (5, 6), suggesting that subclinical cerebrovascular injury may contribute substantially to cognitive deterioration in this population.

Although oral anticoagulation reduces ischemic stroke risk in AF, randomized data have not consistently shown protection against cognitive decline (7). Left atrial appendage occlusion (LAAO) is an established nonpharmacological strategy for stroke prevention in patients at high bleeding risk or unsuitable for long-term anticoagulation (8). However, its impact on cognition remains uncertain, with heterogeneous findings across studies (9, 10). These inconsistent findings may partly reflect differences in baseline cerebrovascular vulnerability among patients undergoing LAAO. These patients are often older, have multiple vascular comorbidities, and frequently harbor pre-existing cerebrovascular injury (11). In this context, procedure-related silent cerebral embolism (SCE) or periprocedural hemodynamic stress may impose additional injury on an already vulnerable brain, thereby influencing postprocedural cognitive trajectories (12, 13).

Cerebral small vessel disease (CSVD) is a common form of covert cerebrovascular injury and an important substrate of cognitive decline (14). On brain magnetic resonance imaging (MRI), CSVD comprises a spectrum of structural abnormalities, including white matter hyperintensities (WMHs), lacunes, cerebral microbleeds (CMBs), and enlarged perivascular spaces (EPVS) (15). The total CSVD burden score integrates multiple MRI markers into a single measure of global microvascular brain injury (16) and may offer incremental prognostic information beyond conventional clinical risk scores. However,whether preprocedural total CSVD burden predicts the longitudinal evolution of cognitive function after LAAO remains unclear.

In this prospective cohort study, we examined whether preprocedural total CSVD burden could identify patients with AF at increased risk of cognitive decline after LAAO. We further explored the prognostic and potential mechanistic relevance of pre-existing microvascular brain injury for one-year cognitive trajectories, with the aim of improving preprocedural risk stratification in this high-risk population.

## Methods

### Study design and participants

This was a prospective, observational cohort study conducted at 2 centers. Consecutive patients with AF who were scheduled to undergo LAAO were enrolled from the Department of Cardiology at the First Affiliated Hospital of Nanjing Medical University and Nantong First People's Hospital between January 2023 and June 2025. Before LAAO, all participants underwent standardized clinical evaluation, cognitive assessment, and brain MRI. Clinical data and cognitive assessments were collected by trained research personnel according to a prespecified protocol. Patients were eligible if they met the following criteria: (1) age 18 years or older; (2) documented AF and planned treatment with LAAO; and (3) completion of preprocedural brain MRI and baseline cognitive assessment. Patients were excluded if they had: (1) neurological disorders that could independently affect cognitive function, including Alzheimer disease, epilepsy, brain tumors, or other major structural brain diseases; (2) psychiatric disorders, severe visual or hearing impairment, aphasia, impaired consciousness, or other conditions precluding reliable cognitive assessment; (3) contraindications to MRI or inability to tolerate MRI acquisition; or (4) recent acute ischemic stroke, intracranial hemorrhage, or other acute intracranial lesions. The study protocol was approved by the institutional ethics committees of the participating centers. Written informed consent was obtained from all participants or their legally authorized representatives. The study was conducted in accordance with the principles of the Declaration of Helsinki.

### Clinical data collection

Baseline demographic and clinical characteristics were collected at the initial in-hospital evaluation. Variables included age, sex, body mass index (BMI), atrial fibrillation (AF) type, paroxysmal AF, and cardiovascular comorbidities, including hypertension, coronary artery disease, diabetes mellitus, prior stroke, and heart failure. Stroke and bleeding risks were assessed using the CHA₂DS₂-VASc and HAS-BLED scores. Echocardiographic parameters, including left atrial diameter (LAD), left ventricular end-diastolic diameter (LVDd), and left ventricular ejection fraction (LVEF), were obtained by routine transthoracic echocardiography.

### Cognitive assessment and outcome definition

Cognitive function was assessed by trained evaluators using standardized procedures in a quiet environment. The Montreal Cognitive Assessment (MoCA) and Mini-Mental State Examination (MMSE), both scored from 0 to 30 with lower scores indicating worse cognitive performance (17, 18), were administered at baseline and at 12 months after LAAO. The MoCA was used as the primary cognitive measure because of its sensitivity for detecting mild cognitive impairment and CSVD-related cognitive dysfunction (19).

The primary outcome was 1-year cognitive decline, defined as a decrease of at least 2 points in the MoCA score from baseline to 12 months (20). This cutoff was selected based on prior studies and was intended to reduce the influence of minor test-retest variability and potential practice effects. In sensitivity analyses, cognitive decline was alternatively defined as a decrease of at least 2 points in the MMSE score.

### Brain MRI acquisition and assessment

Brain MRI was performed at both participating centers using a harmonized standardized protocol on 1.5-T Siemens MRI scanners. The MRI protocol included 3D T1-weighted MPRAGE, 3D T2-weighted SPACE, 3D fluid-attenuated inversion recovery imaging, time-of-flight MR angiography, 3D arterial spin labeling, diffusion-weighted imaging, and susceptibility-weighted imaging. Detailed acquisition parameters are provided in Supplementary Table 1. Diffusion-weighted imaging was used to identify acute ischemic lesions, and susceptibility-weighted imaging was used to detect cerebral microbleeds.

MRI markers of CSVD were assessed according to the Standards for Reporting Vascular Changes on Neuroimaging criteria (14, 15). WMHs were rated using the Fazekas scale. The total CSVD burden score was calculated according to Staals et al. (21), with 1 point assigned for each of the following markers: severe WMHs, defined as a deep WMH Fazekas score of 2 to 3 or a periventricular WMH Fazekas score of 3; at least 1 lacune; moderate-to-severe EPVS in the basal ganglia, defined as ≥11 EPVS; and at least 1 CMB. The total score ranged from 0 to 4, with higher scores indicating greater CSVD burden.

All MRI scans were independently reviewed by 2 experienced neuroradiologists who were blinded to clinical characteristics, procedural information, and cognitive outcomes. Interobserver reliability was assessed using Cohen's kappa, weighted kappa, and intraclass correlation coefficients (ICCs), as appropriate. Disagreements were resolved by consensus or by adjudication from a senior neuroradiologist. As shown in Supplementary Table 2, interobserver agreement was substantial for severe WMH presence, lacune presence, CMB presence, and moderate-to-severe basal ganglia EPVS, with kappa values of 0.84, 0.81, 0.70, and 0.84, respectively. Agreement for the total CSVD burden score was also substantial, with a weighted kappa of 0.74 and an ICC of 0.90, with a 95% confidence interval of 0.86 to 0.93.

SCE was defined as a new diffusion-restricted ischemic lesion on follow-up MRI, detected on diffusion-weighted imaging, in the absence of corresponding focal neurological symptoms or a clinical diagnosis of stroke. For the primary analyses, postprocedural SCE was coded as a cumulative binary variable, defined as the occurrence of at least one SCE on any scheduled follow-up MRI from the early postprocedural scan through the 12-month follow-up.

### Follow-up

All participants underwent baseline clinical evaluation, cognitive assessment, and preprocedural brain MRI before LAAO. Follow-up brain MRI was scheduled early after LAAO and at 3, 6, and 12 months according to the study protocol. Cognitive function was reassessed at 12 months after the procedure. The 12-month cognitive assessment and ascertainment of key clinical outcomes were performed in the outpatient setting by trained research personnel who were blinded to procedural details and baseline MRI findings.

## Statistical analysis

Continuous variables are presented as mean ± standard deviation or median with interquartile range, as appropriate, and categorical variables are presented as counts and percentages. Baseline characteristics were compared between patients with and without 1-year cognitive decline using the Student's t test or Mann–Whitney U test for continuous variables and the chi-square test or Fisher's exact test for categorical variables, as appropriate.

The primary endpoint was 1-year cognitive decline, defined as a decrease of at least 2 points in the Montreal Cognitive Assessment score from baseline to 12 months after left atrial appendage occlusion. The primary exposure was the preprocedural total CSVD burden score. Logistic regression was used to evaluate the association between CSVD burden and 1-year MoCA-defined cognitive decline. Model 1 was unadjusted, and Model 2 was adjusted for age and sex. To reduce the risk of overfitting given the limited number of outcome events, Model 3 was constructed as a LASSO-informed parsimonious multivariable model rather than as a broad fully adjusted model. Candidate variables for least absolute shrinkage and selection operator (LASSO) were restricted to clinically relevant preprocedural variables, including age, sex, BMI, paroxysmal AF, hypertension, coronary artery disease, diabetes mellitus, prior stroke, heart failure, baseline MoCA score, CHA_2_DS_2_-VASc score, HAS-BLED score, LAD, LVDd, LVEF, and total CSVD burden score. Postprocedural SCE was not included in the LASSO procedure or in the preprocedural prediction model because it occurred after LAAO. LASSO logistic regression was performed using 10-fold cross-validation, and variables with nonzero coefficients at the value of lambda that minimized the cross-validated error, *λ*min, were retained. Total CSVD burden score was included as one of the candidate predictors and was selected by LASSO, rather than being forced into the model. Hypertension and baseline MoCA score were also retained by LASSO-based variable reduction. Age and sex were forced into the final multivariable model *a priori* because of their clinical relevance, although they were not selected by LASSO. Therefore, Model 3 included age, sex, hypertension, baseline MoCA score, and total CSVD burden score. Odds ratios (ORs) are presented per 1-point increase in the total CSVD burden score. Firth's penalized logistic regression using the same covariate set as Model 3 was performed as a sensitivity analysis to reduce potential sparse-data and small-sample bias. An additional sensitivity analysis was performed by forcing prior stroke into the Firth penalized logistic regression model.

Based on this LASSO-informed parsimonious covariate set, two nested preprocedural prediction models were compared to evaluate the incremental predictive value of CSVD burden. The clinical model included age, sex, hypertension, and baseline MoCA score. The extended model additionally included the total CSVD burden score and therefore corresponded to the parsimonious Model 3 used in the association analysis. Model discrimination was evaluated using the area under the receiver operating characteristic curve (AUC). The DeLong test was used to compare apparent AUCs between nested models. Calibration was assessed using calibration plots and calibration slope. Overall prediction error was assessed using the Brier score. Clinical usefulness was evaluated using decision curve analysis by comparing the net benefit of the clinical model and the clinical plus CSVD burden model across a range of threshold probabilities. Internal validation of the final prediction models was performed using 1,000 bootstrap resamples. In the primary fixed-model bootstrap validation, the final predictor sets were kept fixed, whereas model coefficients were re-estimated in each bootstrap sample to estimate optimism in model performance. These fixed-model bootstrap validation results were used as the primary internal validation estimates and are reported in the main tables. Optimism-corrected performance was calculated by subtracting the average bootstrap-estimated optimism from the apparent performance.

To further account for potential overfitting and uncertainty introduced by variable selection, a sensitivity full-process bootstrap validation was additionally performed for the preprocedural clinical plus CSVD burden model. In each bootstrap resample, the entire model-building process was repeated, including 10-fold cross-validated LASSO variable selection at *λ*min, forced inclusion of age and sex, and refitting of the multivariable logistic regression model. Variable selection frequencies were calculated across bootstrap resamples. A nomogram was constructed based on the final preprocedural clinical plus CSVD burden model.

Mediation analysis was performed to explore whether postprocedural SCE mediated the association between preprocedural CSVD burden and 1-year cognitive decline. The average causal mediation effect, average direct effect, total effect, and proportion mediated were estimated using bootstrap resampling with 1,000 simulations. We also examined the individual pathways from CSVD burden to SCE and from SCE to cognitive decline after adjustment for CSVD burden and relevant covariates. Mediation analysis assumes correct temporal ordering, no unmeasured confounding, and correct model specification. Because SCE occurred after LAAO, it was not included in the preprocedural prediction model. Its incremental value was evaluated separately.

Sensitivity analysis was performed using an alternative cognitive outcome defined as a ≥2-point decrease in MMSE score. Missing data were handled using multiple imputation by chained equations, with 5 imputed datasets generated and estimates combined using Rubin's rules when appropriate. All tests were 2-sided, and *P* < 0.05 was considered statistically significant. Analyses were performed using R software.

## Results

### Patient characteristics

A total of 110 patients were included in the analysis (Figure 1). The mean age was 69.10 ± 6.48 years, and 71 patients were men (64.5%). The median total CSVD burden score was 1.50 [1.00, 2.00]. Postprocedural SCE was detected in 42 patients (38.2%), and 32 patients (29.1%) experienced cognitive decline during 1-year follow-up. Perioperative SCE was detected on early postprocedural MRI in 35 patients, and newly detected SCEs were observed in 22 patients during later follow-up MRI at 3, 6, or 12 months; some patients had SCEs detected at more than one time point. Baseline and postprocedural characteristics are shown in Table 1. Compared with patients without cognitive decline, those with cognitive decline had a higher prevalence of WMH, lacunes, CMBs, EPVS, and SCE, as well as a higher total CSVD burden score. Age, sex, BMI, most vascular comorbidities, risk scores, cardiac parameters, and baseline cognitive scores were similar between groups.

**Figure 1:**
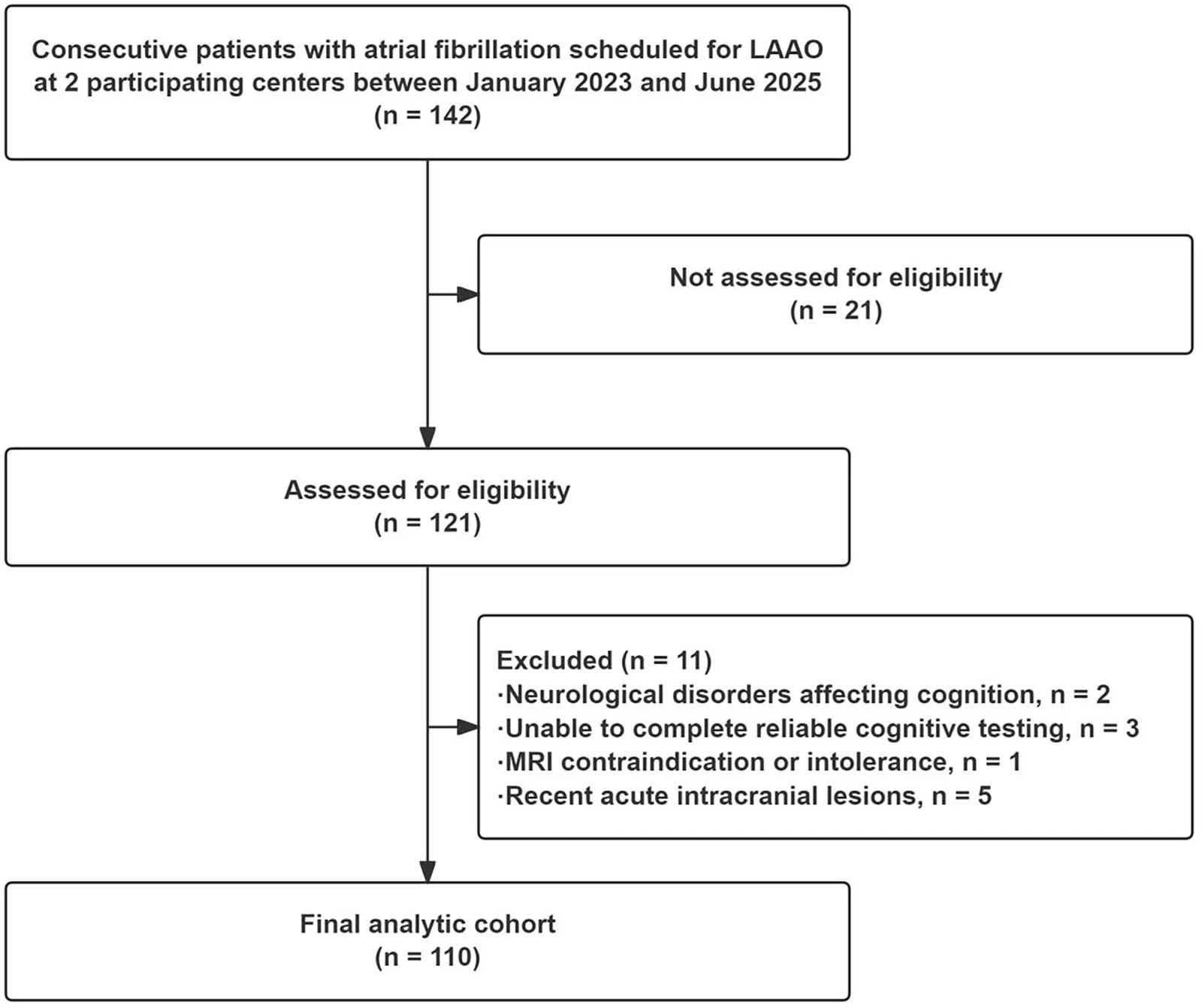
Study flow diagram.

**Table 1:** Baseline, postprocedural, and follow-up characteristics according to 1-year cognitive decline.

Variable	Overall	Group		*P* value
		No cognitive decline	Cognitive decline	
*n*	110	78	32	
Age, years	69.10 ± 6.48	68.82 ± 6.85	69.78 ± 5.51	0.482
Male, *n* (%)	71 (64.5)	50 (64.1)	21 (65.6)	1.000
BMI, kg/m2	24.35 ± 2.52	24.33 ± 2.55	24.41 ± 2.48	0.879
PAF, *n* (%)	43 (39.1)	31 (39.7)	12 (37.5)	0.997
Hypertension, *n* (%)	74 (67.3)	48 (61.5)	26 (81.2)	0.076
CAD, *n* (%)	26 (23.6)	18 (23.1)	8 (25.0)	1.000
DM, *n* (%)	32 (29.1)	23 (29.5)	9 (28.1)	1.000
Prior stroke, *n* (%)	89 (80.9)	64 (82.1)	25 (78.1)	0.835
HF, *n* (%)	6 (5.5)	4 (5.1)	2 (6.2)	1.000
CHA₂DS₂-VASc score	4.00 [4.00, 5.00]	4.00 [4.00, 5.00]	4.00 [4.00, 5.00]	0.889
HAS-BLED score	2.00 [2.00, 3.00]	2.00 [2.00, 3.00]	3.00 [2.00, 3.00]	0.089
LAD, mm	45.25 ± 4.53	45.16 ± 4.60	45.47 ± 4.43	0.743
LVDd, mm	48.29 ± 3.97	48.53 ± 4.12	47.69 ± 3.58	0.316
LVEF, %	61.99 ± 4.78	61.89 ± 5.24	62.23 ± 3.46	0.732
WMH, *n* (%)	37 (33.6)	18 (23.1)	19 (59.4)	0.001
Lacunes, *n* (%)	80 (72.7)	49 (62.8)	31 (96.9)	0.001
CMBs, *n* (%)	19 (17.3)	5 (6.4)	14 (43.8)	<0.001
EPVS, *n* (%)	37 (33.6)	20 (25.6)	17 (53.1)	0.011
CSVD burden score	1.50 [1.00, 2.00]	1.00 [0.25, 2.00]	2.50 [2.00, 3.00]	<0.001
SCE, *n* (%)	42 (38.2)	18 (23.1)	24 (75.0)	<0.001
MMSE at baseline	24.90 ± 3.61	24.96 ± 3.45	24.75 ± 4.02	0.781
MMSE at 1 year	24.75 ± 5.01	25.96 ± 3.79	21.78 ± 6.31	<0.001
MoCA at baseline	19.89 ± 4.45	19.62 ± 4.43	20.56 ± 4.51	0.313
MoCA at 1 year	20.13 ± 5.69	21.76 ± 5.01	16.16 ± 5.36	<0.001

Values are presented as mean ± SD, median [IQR], or *n* (%) as appropriate.

BMI, body mass index; CAD, coronary artery disease; CMBs, cerebral microbleeds; CSVD, cerebral small vessel disease; DM, diabetes mellitus; EPVS, enlarged perivascular spaces; HF, heart failure; LAD, left atrial diameter; LAAO, left atrial appendage occlusion; LVDd, left ventricular end-diastolic diameter; LVEF, left ventricular ejection fraction; MMSE, mini-mental state examination; MoCA, montreal cognitive assessment; PAF, paroxysmal atrial fibrillation; SCE, silent cerebral embolism; WMH, white matter hyperintensity.

### Independent association with cognitive decline and risk model development

Logistic regression analyses showed that a higher total CSVD burden score was significantly associated with increased odds of 1-year MoCA-defined cognitive decline (Table 2). This association was observed in the unadjusted model (OR: 4.79; 95% CI: 2.73–9.51; *P* < 0.001) and after adjustment for age and sex (OR: 4.99; 95% CI: 2.80–10.19; *P* < 0.001).

**Table 2:** Association between total CSVD burden score and 1-year cognitive decline.

Model	OR (95% CI)	*P* value
Model 1	4.79 (2.73−9.51)	<0.001
Model 2	4.99 (2.80–10.19)	<0.001
Model 3	5.39 (2.91–11.75)	<0.001

ORs are presented for each 1-point increase in the total CSVD burden score. Model 1 was unadjusted. Model 2 was adjusted for age and sex. Model 3 was a LASSO-informed parsimonious multivariable model that included total CSVD burden score, age, sex, hypertension, and baseline MoCA score. Total CSVD burden score, hypertension, and baseline MoCA score were retained by LASSO, whereas age and sex were prespecified and forced into the model because of their clinical relevance.

CI, confidence interval; CSVD, cerebral small vessel disease; MoCA, montreal cognitive assessment; OR, odds ratio.

To develop a parsimonious preprocedural prediction model, 10-fold cross-validated LASSO logistic regression was performed using clinically relevant variables available before the procedure. At *λ*min, total CSVD burden score, hypertension, and baseline MoCA score were retained as predictors with nonzero coefficients (Figure 2). Thus, total CSVD burden score was selected by LASSO rather than forced into the model. Although age and sex were not selected by LASSO, they were prespecified and retained because of their clinical relevance. Accordingly, the final LASSO-informed parsimonious model included age, sex, hypertension, baseline MoCA score, and total CSVD burden score. In this LASSO-informed parsimonious multivariable model, total CSVD burden score remained associated with 1-year MoCA-defined cognitive decline (OR: 5.39; 95% CI: 2.91–11.75; *P* < 0.001). In a sensitivity analysis using Firth penalized logistic regression with the same covariates, the association between total CSVD burden score and cognitive decline remained significant, although the effect estimate was slightly attenuated (OR: 4.61; 95% CI: 2.60–9.48; *P* < 0.001; Supplementary Table 3). In an additional sensitivity analysis further forcing prior stroke into the model, total CSVD burden score remained significantly associated with cognitive decline (OR: 4.53 per 1-point increase; 95% CI: 2.57–9.24; *P* < 0.001).

**Figure 2:**
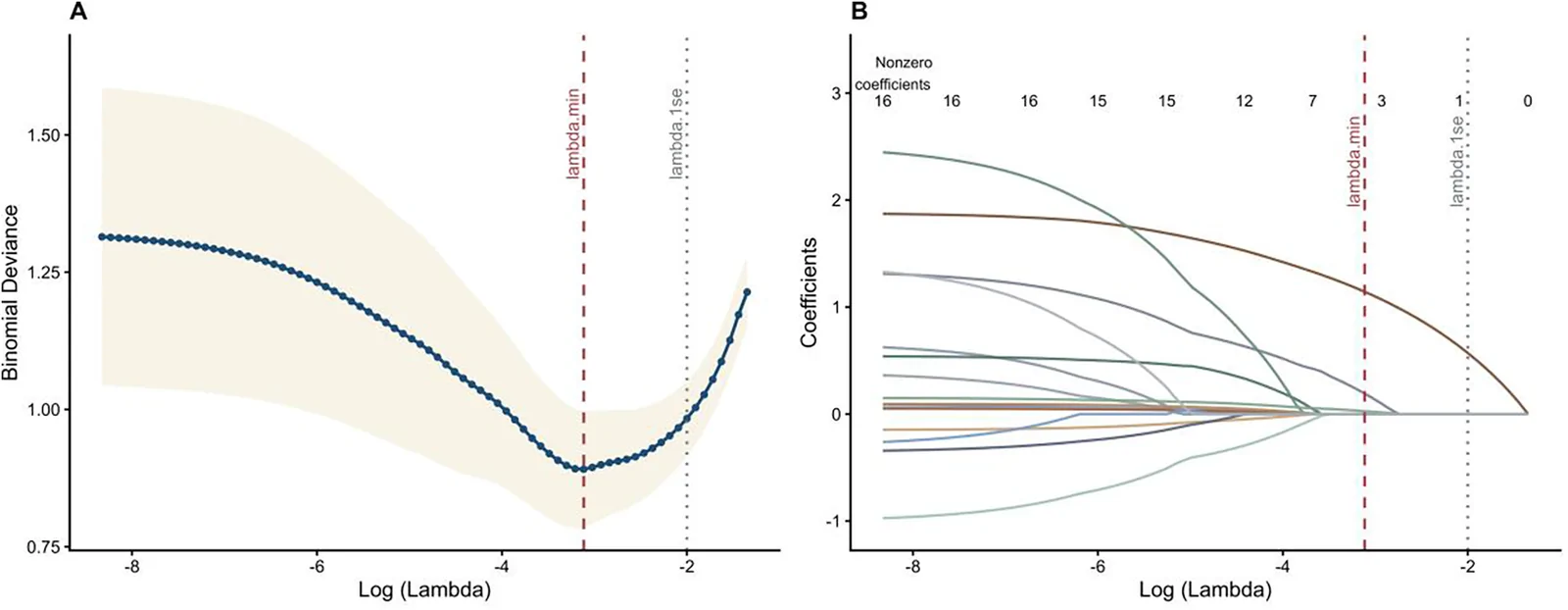
Predictor selection using LASSO logistic regression. **(A)** Ten-fold cross-validation was used to select the optimal tuning parameter in the LASSO logistic regression model. The red dashed line indicates *λ*_min, and the gray dotted line indicates *λ*_1se. **(B)** Variables with nonzero coefficients at *λ*_min were identified by LASSO; prespecified clinical variables were additionally retained in the final model. LASSO, least absolute shrinkage and selection operator.

In the primary fixed-model bootstrap validation, the clinical plus CSVD burden model showed better predictive performance than the clinical model, with the optimism-corrected AUC increasing from 0.608 to 0.863 and the apparent Brier score decreasing from 0.194 to 0.128 (Table 3). Calibration plots further demonstrated better agreement between predicted and observed risks after inclusion of total CSVD burden score (Figure 3). Decision curve analysis showed that the clinical plus total CSVD burden model achieved greater net benefit than the clinical model across a wide range of threshold probabilities, suggesting improved usefulness for risk stratification (Supplementary Figure 1).

**Table 3:** Performance of preprocedural models for predicting 1-year MoCA-defined cognitive decline.

Metric	Clinical model	Clinical + CSVD burden model
Apparent AUC	0.670	0.890
Optimism-corrected AUC	0.608	0.863
Apparent Brier score	0.194	0.128
Calibration slope, optimism-corrected	0.649	0.836
DeLong test for AUC comparison, *P* value	—	<0.001
Likelihood ratio test, *P* value	—	<0.001

The clinical model included age, sex, hypertension, and baseline MoCA score. The clinical plus CSVD burden model additionally included the total CSVD burden score. Internal validation was performed using 1,000 bootstrap resamples with the final predictor sets kept fixed. The optimism-corrected AUC and calibration slope were calculated from fixed-model bootstrap validation. Apparent Brier scores were calculated from apparent predicted probabilities and are reported descriptively. The DeLong test compared apparent AUCs between models, and the likelihood ratio test assessed improvement in model fit between nested logistic regression models. A sensitivity full-process bootstrap validation with repeated LASSO variable selection is reported in Supplementary Table 4.

AUC, area under the receiver-operating characteristic curve; CSVD, cerebral small vessel disease; MoCA, montreal cognitive assessment.

**Figure 3:**
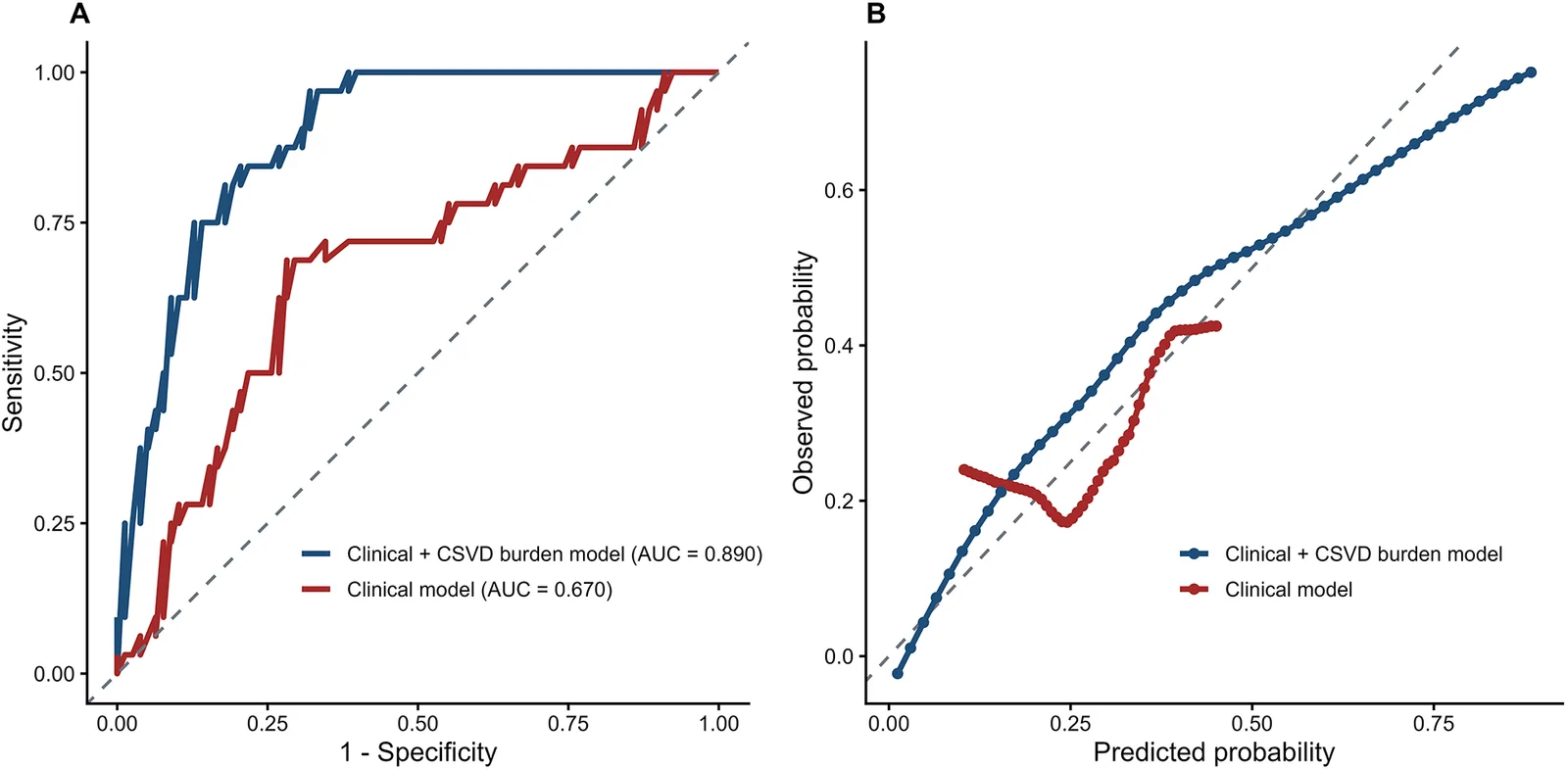
Incremental predictive value of CSVD burden score for 1-year cognitive decline. **(A)** Apparent receiver-operating characteristic curves comparing the clinical model with the clinical plus CSVD burden model. The apparent AUCs are shown in the legend. In fixed-model bootstrap internal validation, addition of CSVD burden improved the optimism-corrected AUC from 0.608 to 0.863. **(B)** Calibration curves for the clinical model and the clinical plus CSVD burden model. The dashed diagonal line indicates perfect calibration. AUC, area under the receiver-operating characteristic curve; CSVD, cerebral small vessel disease.

To further assess potential overfitting related to variable selection, a more stringent full-process bootstrap sensitivity analysis was performed. In each bootstrap resample, the entire model-building process was repeated, including 10-fold cross-validated LASSO variable selection at *λ*min, forced inclusion of age and sex, and model refitting. In this analysis, the clinical plus CSVD burden model had an apparent AUC of 0.890 and an optimism-corrected AUC of 0.810. The apparent Brier score was 0.128 and increased to 0.184 after optimism correction, and the optimism-corrected calibration slope was 0.518, suggesting some degree of overfitting and overly extreme predicted risks (Supplementary Table 4).

During full-process bootstrap validation, total CSVD burden score was selected in 100.0% of resamples, whereas baseline MoCA score and hypertension were selected in 74.0% and 70.4% of resamples, respectively. Other candidate variables showed lower or moderate selection frequencies and were not retained in the original LASSO model. Age and sex were forced into all models *a priori* and were therefore not considered LASSO-selected variables. These findings support the robustness of total CSVD burden score as a predictor, while indicating that the prediction model should be interpreted cautiously and requires external validation before clinical implementation (Supplementary Table 5). To facilitate individualized prediction of 1-year cognitive decline, a nomogram incorporating total CSVD burden score, hypertension, baseline MoCA score, age, and sex was constructed and is shown in Figure 4.

**Figure 4:**
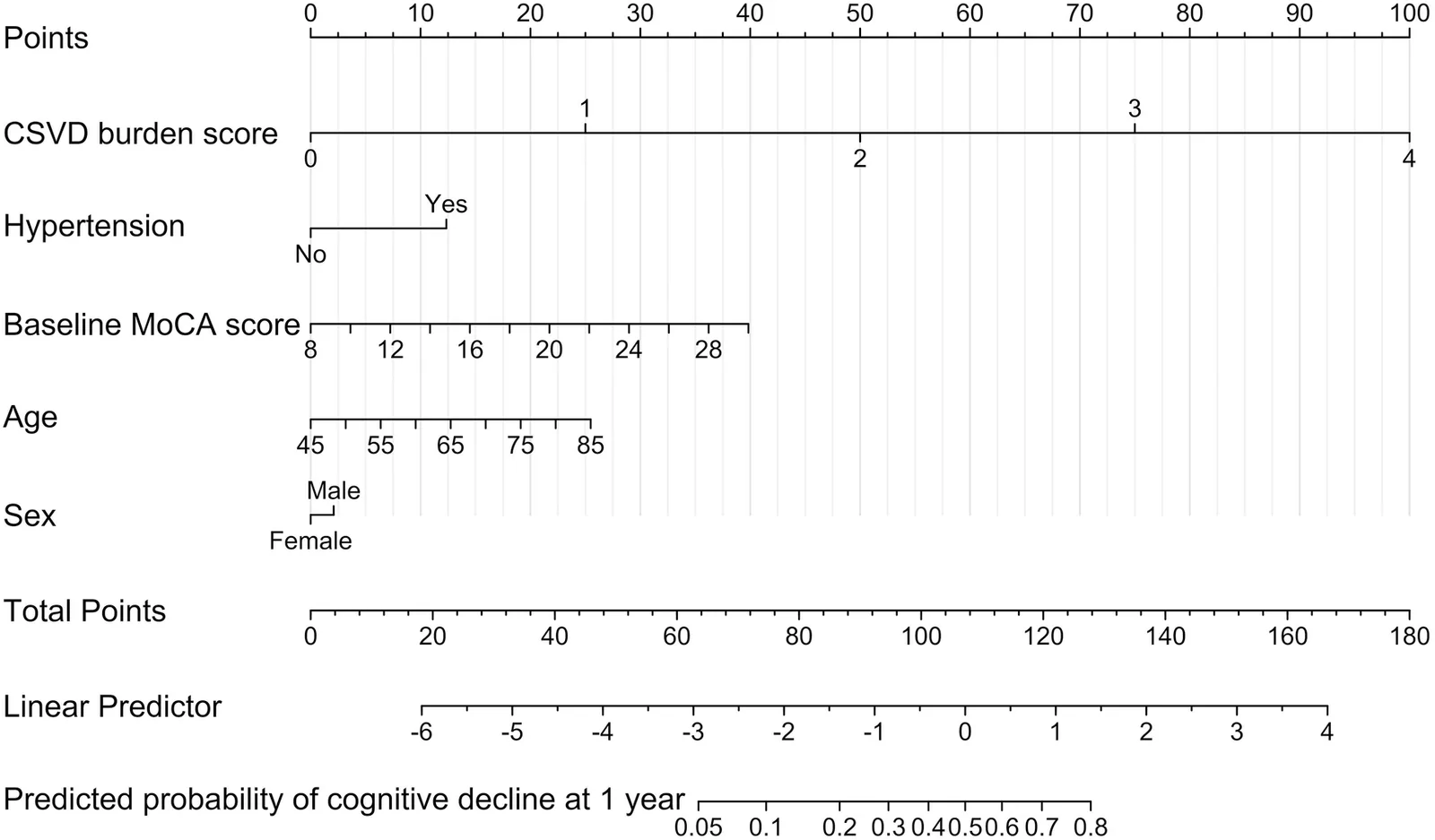
Nomogram for predicting 1-year MoCA-defined cognitive decline. The nomogram estimates the probability of 1-year cognitive decline after LAAO based on CSVD burden score, hypertension, baseline MoCA score, age, and sex. Points assigned to each predictor are summed to obtain the total points, which correspond to the predicted probability of cognitive decline at 1 year. CSVD, cerebral small vessel disease; LAAO, left atrial appendage occlusion; MoCA, montreal cognitive assessment.

### Mediation and incremental predictive value of SCE

In mediation analysis adjusted for age, sex, hypertension, and baseline MoCA score, Exploratory mediation analysis suggested that SCE may partly account for the association between total CSVD burden score and 1-year cognitive decline. The estimated proportion mediated was 28.3%, although the confidence interval for the indirect effect was wide (Table 4). In fixed-model bootstrap validation, adding SCE to the model containing total CSVD burden score and clinical covariates modestly improved discrimination (Table 5 and Figure 5), with the apparent AUC increasing from 0.890 to 0.902 and the optimism-corrected AUC from 0.863 to 0.873. The AUC increase was not statistically significant by the DeLong test, but model fit improved significantly according to the likelihood ratio test. The postprocedural model including SCE also had a lower apparent Brier score, although the optimism-corrected calibration slope slightly decreased. These findings suggest that SCE may partly mediate the association between total CSVD burden score and cognitive decline, while providing complementary, although limited, predictive information. In a sensitivity mediation analysis using only perioperative SCE detected on early postprocedural MRI as the mediator, the indirect effect remained significant (ACME = 0.0137, 95% CI: 0.0003–0.0300; *P* = 0.040; Supplementary Table 6).

**Table 4:** Mediation analysis of the association between total CSVD burden score and 1-year MoCA-defined cognitive decline through SCE.

Effect	Type	Estimate	95% CI	*P* value
CSVD burden score → SCE	OR	3.49	2.17–6.11	<0.001
SCE → cognitive decline	OR	4.70	1.45–16.39	0.011
Total effect	Effect size	0.0810	0.0324–0.1200	<0.001
ACME	Effect size	0.0229	0.0002–0.0400	0.044
ADE	Effect size	0.0581	0.0240–0.1000	<0.001
Proportion mediated	%	28.3%	0.5%–46.0%	0.044

Mediation analysis was performed to assess whether SCE mediated the association between preprocedural CSVD burden score and 1-year MoCA-defined cognitive decline. All models were adjusted for age, sex, hypertension, and baseline MoCA score. Estimates for the total effect, ACME, and ADE represent absolute changes in the probability of cognitive decline. Values are presented as odds ratios (ORs) for logistic regression paths and as effect estimates for mediation analysis.

ACME, average causal mediation effect; ADE, average direct effect; CI, confidence interval; CSVD, cerebral small vessel disease; MoCA, montreal cognitive assessment; SCE, silent cerebral embolism.

**Table 5:** Incremental predictive value of postprocedural SCE beyond the clinical plus CSVD burden model for 1-year MoCA-defined cognitive decline.

Metric	Clinical + CSVD burden model	Clinical + CSVD burden model + SCE
Apparent AUC	0.890	0.902
Optimism-corrected AUC	0.863	0.873
Apparent Brier score	0.128	0.115
Optimism-corrected calibration slope	0.836	0.810
DeLong test for AUC comparison, *P* value	—	0.463
Likelihood ratio test, *P* value	—	0.010

The clinical plus CSVD burden model included age, sex, hypertension, baseline MoCA score, and total CSVD burden score. The postprocedural model additionally included SCE. Internal validation was performed using 1,000 bootstrap resamples. The DeLong test compared apparent AUCs between models, and the likelihood ratio test assessed improvement in model fit between nested logistic regression models.

AUC, area under the receiver-operating characteristic curve; CSVD, cerebral small vessel disease; MoCA, montreal cognitive assessment; SCE, silent cerebral embolism.

**Figure 5:**
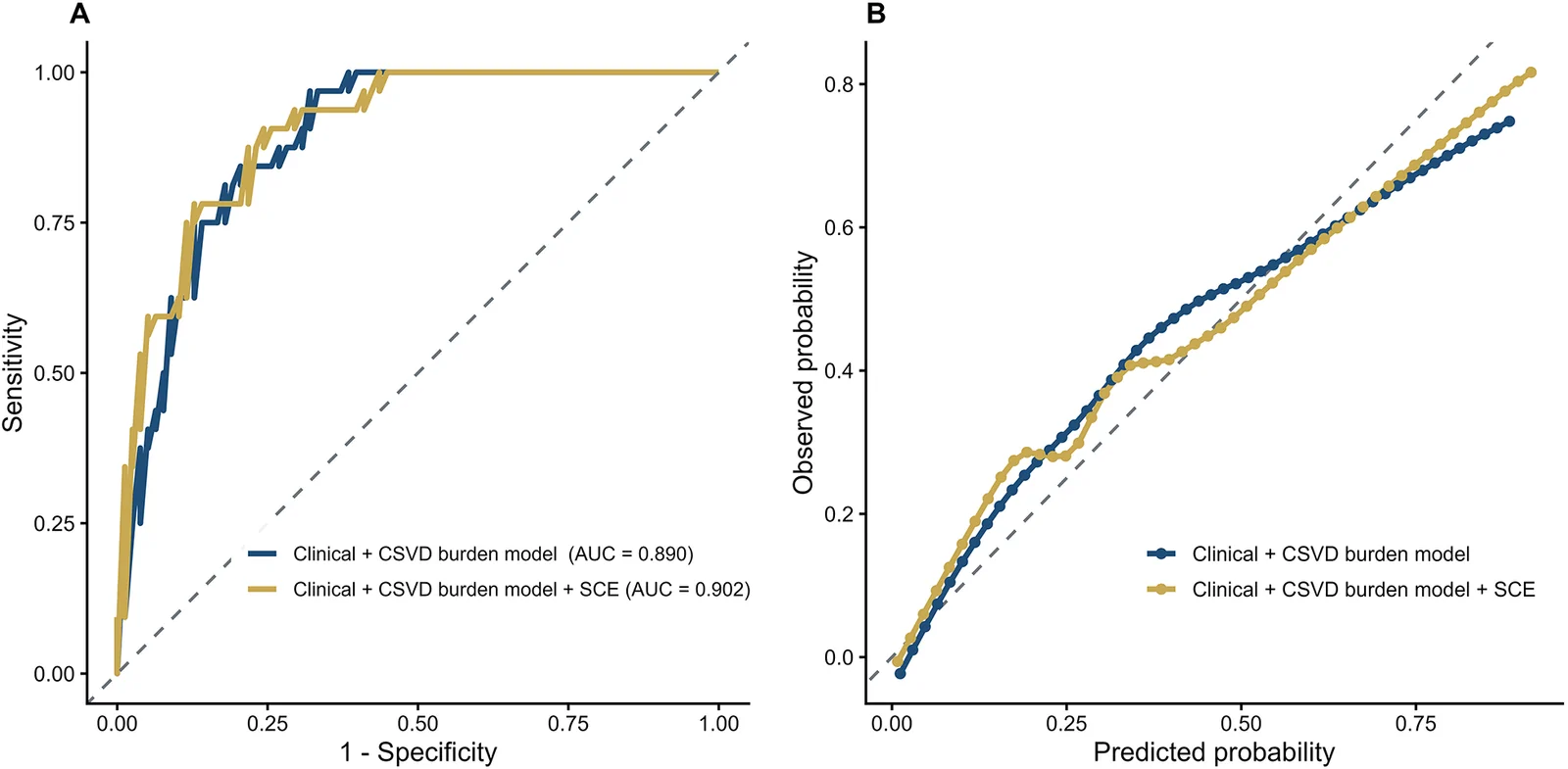
Predictive performance after adding SCE to the clinical plus CSVD burden model. **(A)** Apparent receiver-operating characteristic curves comparing the clinical plus CSVD burden model with the extended model additionally including SCE. The apparent AUC increased from 0.890 to 0.902 after addition of SCE; after bootstrap optimism correction, the AUC increased from 0.863 to 0.873. **(B)** Calibration curves for both models. The dashed diagonal line indicates perfect calibration. AUC, area under the receiver-operating characteristic curve; CSVD, cerebral small vessel disease; SCE, silent cerebral embolism.

### Sensitivity analyses

In sensitivity analyses using 1-year MMSE decline of at least 2 points as an alternative cognitive outcome, the results were broadly consistent with the primary MoCA-based findings. After adjustment for age, sex, hypertension, and baseline MMSE score, total CSVD burden score remained significantly associated with MMSE-defined cognitive decline. In the MMSE-based mediation analysis, total CSVD burden score showed a significant direct association with MMSE-defined cognitive decline, whereas the indirect effect through SCE was not statistically significant (Supplementary Table 7). For prediction, adding total CSVD burden score to the clinical model markedly improved discrimination and model fit, increasing the optimism-corrected AUC from 0.609 to 0.856. Further addition of SCE yielded modest incremental improvement, with an optimism-corrected AUC of 0.866 and a lower Brier score; the AUC improvement was not statistically significant, although model fit improved according to the likelihood ratio test (Supplementary Table 8).

## Discussion

In this prospective cohort of patients with AF undergoing LAAO, preprocedural total CSVD burden was independently associated with 1-year cognitive decline and substantially improved risk prediction beyond clinical variables and baseline cognitive performance. These findings suggest that MRI-defined microvascular brain injury captures cerebral vulnerability not adequately reflected by conventional cardiovascular risk scores. Importantly, CSVD burden should not be interpreted as a reason to withhold LAAO. Rather, it may help identify patients who require individualized counseling, optimized procedural planning, and closer cognitive follow-up.

As the use of LAAO continues to expand, postprocedural cognitive outcomes are becoming increasingly relevant (22). The clinical value of LAAO has traditionally been assessed by its ability to reduce overt stroke, systemic embolism, and bleeding events (23, 24). However, covert brain injury and cognitive outcomes have received less attention, which may be particularly relevant in older patients with high vascular risk. Left atrial instrumentation and device deployment may generate SCE (25), and our previous work suggested that SCE after LAAO is associated with subsequent cognitive decline (10). However, because SCE occurs after the procedure, it cannot be used for preprocedural risk stratification. This highlights the need for a preprocedural marker that identifies patients who are cognitively vulnerable before LAAO. In the present study, preprocedural CSVD burden addressed this gap: it was associated with subsequent cognitive decline and improved risk prediction beyond traditional clinical variables and baseline cognition. These findings support CSVD burden as a preprocedural marker of cerebral vulnerability in patients considered for LAAO.

SCE may provide supportive, hypothesis-generating mechanistic information. The mediation analysis was compatible with the possibility that SCE partly contributes to the association between preprocedural CSVD burden and 1-year cognitive decline. Importantly, the direct effect of CSVD burden remained significant after accounting for SCE, indicating partial rather than complete mediation. These findings suggest that procedure-related embolic injury may represent one pathway linking baseline cerebral vulnerability to post-LAAO cognitive deterioration, but it does not fully explain this association (9). Patients with higher CSVD burden may be more susceptible to SCE after LAAO, but the significant direct effect indicates that SCE accounts for only part of the subsequent cognitive risk (26). Consistent with this interpretation, adding SCE only modestly improved discrimination beyond CSVD burden and clinical variables. Thus, SCE may help clarify one biological pathway, whereas CSVD burden provides broader and more clinically useful information for preprocedural risk stratification.

These findings suggest that postprocedural brain injury is not determined solely by the cardiac embolic substrate or traditional clinical risk profile. Rather, the baseline vulnerability of the brain may influence whether a small embolic or hemodynamic insult becomes an MRI-detectable lesion (15, 27). Total CSVD burden may therefore be viewed as an imaging phenotype of a “vulnerable brain.” CSVD reflects chronic microvascular injury, including endothelial dysfunction, blood-brain barrier disruption, impaired vasoreactivity, chronic hypoperfusion, and reduced cerebrovascular reserve (28, 29). Patients with AF frequently exhibit a high burden of these lesions, together with clinically overt and covert ischemic infarcts (30, 31). These changes may reduce cerebrovascular reserve and lower the threshold for DWI-positive lesions after transient ischemia or microembolism. Thus, similar procedural insults during LAAO may be more likely to manifest as SCE in patients with higher CSVD burden, whereas patients with lower CSVD burden may better tolerate such insults through preserved autoregulation and collateral flow.

Sensitivity analyses using MMSE decline yielded findings broadly consistent with the primary MoCA-based results. The indirect effect through SCE was directionally consistent but did not reach statistical significance for MMSE decline, although the direction and proportion mediated were comparable. This difference may reflect the lower sensitivity of MMSE for detecting subtle vascular cognitive changes, particularly in executive and attentional domains, which are better captured by MoCA (32). These findings support the robustness of the main findings while reinforcing MoCA as the more sensitive measure for post-LAAO cognitive change.

Several limitations should be acknowledged. First, this was an observational study, and residual confounding cannot be excluded despite multivariable adjustment. Second, the sample size and number of cognitive events were limited, which may have reduced statistical power, particularly in mediation and incremental prediction analyses. Although a full-process bootstrap validation, including repeated LASSO variable selection, suggested that total CSVD burden score was a robust predictor, the optimism-corrected calibration slope indicated some degree of overfitting; therefore, the model should be interpreted cautiously and requires external validation. In addition, because the clinical model was derived using LASSO, hypertension was retained as the strongest predictor in this cohort, whereas other vascular risk factors such as diabetes and prior stroke did not contribute additional independent information after penalization. Nevertheless, prior stroke remains a potential confounder, and although additional adjustment for prior stroke did not materially alter the association between total CSVD burden and cognitive decline, residual confounding by stroke-related characteristics cannot be excluded. Residual confounding from other baseline factors, such as functional status and psychiatric history, may also have influenced cognitive outcomes. Third, although SCE was assessed using serial MRI, lesion volume, location, and evolution were not fully characterized, and SCE detected at different follow-up intervals may reflect heterogeneous mechanisms. Therefore, the cumulative SCE variable should be interpreted as an overall postprocedural SCE measure rather than as a purely perioperative procedural injury marker. Fourth, cognitive function was evaluated using MoCA and MMSE rather than comprehensive neuropsychological batteries. Although the ≥2-point MoCA threshold was selected *a priori* to capture cognitive worsening more likely to exceed minor test-retest variability and practice effects, a universally accepted minimal clinically important difference for MoCA has not been established in patients undergoing LAAO. Nevertheless, the use of both MoCA and MMSE enabled sensitivity analyses, and the generally consistent direction of findings strengthened the robustness of our results. Finally, mediation analysis relies on assumptions regarding temporality, no unmeasured confounding, and correct model specification; therefore, the mediation findings should be interpreted as supportive mechanistic evidence rather than proof of causality. Although decision curve analysis suggested incremental net benefit, these findings reflect predictive performance in a single-cohort internal validation setting and should not yet be interpreted as evidence of established clinical utility. External validation and prospective impact studies are needed before implementation.

## Conclusions

Preprocedural total CSVD burden score was associated with 1-year cognitive decline after LAAO and improved prognostic discrimination beyond clinical variables. SCE may partly mediate this association, supporting total CSVD burden score as a potential marker of vulnerability to postprocedural cognitive decline.

## Data Availability

The raw data supporting the conclusions of this article will be made available by the authors, without undue reservation.
