# Supplementary material for: Preprocedural cerebral small vessel disease burden predicts one-year cognitive decline in patients with atrial fibrillation undergoing left atrial appendage occlusion: a prediction model and mediation analysis
Source: Front Cardiovasc Med. 2026 Oct 30;13:1948991. doi: 10.3389/fcvm.2026.1948991 (PMC13759352; doi:10.3389/fcvm.2026.1948991)
Supplement: Supplementary file 1 [file Supplementaryfile1.docx]

Supplementary Table 1. Brain MRI acquisition parameters.

| **Sequence** | **Plane** | **Voxel size (mm)** | **Slice thickness (mm)** | **TR (ms)** | **TE (ms)** | **Key parameters** | **Scan time** |
| --- | --- | --- | --- | --- | --- | --- | --- |
| 3D T1-MPRAGE | Sagittal | 1.0 × 1.0 × 1.0 | 1 | 1900 | 2.45 | 3D volume; A>>P; 1 average; 176 slices/slab | 3:55 |
| 3D T2-SPACE | Sagittal | 0.9 × 0.9 × 0.9 | 0.9 | 3200 | 408 | 3D volume; A>>P; 1.4 averages; 192 slices/slab | 3:23 |
| 3D T2-FLAIR | Sagittal | 0.9 × 0.9 × 1.0 | 1 | 5000 | 381 | 3D volume; A>>P; 1 average; 160 slices/slab | 4:35 |
| TOF-MRA | Axial | 0.3 × 0.3 × 0.6 | 0.6 | 21 | 3.42 | 4 slabs; R>>L; 6/8 partial Fourier; 1 average | 4:10 |
| 3D-ASL | Axial | 1.5 × 1.5 × 3.0 | 3 | 4300 | 16.48 | PLD = 2000 ms; R>>L; 16 averages; phase oversampling 30% | 3:31 |
| DWI | Axial | 0.6 × 0.6 × 5.0 | 5 | 6400 | 98 | b = 0 and 1000 s/mm²; 3 diffusion directions; 2 averages/b-value | 1:12 |
| SWI | Axial | 0.6 × 0.6 × 2.0 | 2 | 28 | 20 | A>>P; 1 average; 7/8 partial Fourier; normalize filter | 2:19 |

Supplementary Table 2. Interobserver reliability of CSVD imaging markers.

| Imaging marker | Statistic | Reader 1 vs Reader 2 |
| --- | --- | --- |
| Severe WMH presence | Cohen’s kappa | 0.84 |
| Lacune presence | Cohen’s kappa | 0.81 |
| CMB presence | Cohen’s kappa | 0.70 |
| Moderate-to-severe basal ganglia EPVS | Cohen’s kappa | 0.84 |
| Total CSVD burden score | Weighted kappa | 0.74 |
| Total CSVD burden score | ICC | 0.90 (95% CI, 0.86–0.93) |

Abbreviations: CMB, cerebral microbleed; CSVD, cerebral small vessel disease; EPVS, enlarged perivascular spaces; ICC, intraclass correlation coefficient; WMH, white matter hyperintensity.

Supplementary Table 3. Conventional and Firth penalized logistic regression analyses for the association between total CSVD burden and 1-year MoCA-defined cognitive decline.

| **Variable** | **Conventional logistic regression** | | **Firth penalized logistic regression** | |
| --- | --- | --- | --- | --- |
|  | **OR (95% CI)** | **P value** | **OR (95% CI)** | **P value** |
| CSVD burden score | 5.39 (2.91–11.75) | <0.001 | 4.61 (2.60–9.48) | <0.001 |
| Hypertension | 2.30 (0.72–8.21) | 0.175 | 2.08 (0.69–6.92) | 0.196 |
| Baseline MoCA score | 1.13 (0.98–1.31) | 0.090 | 1.12 (0.98–1.28) | 0.096 |
| Age | 1.04 (0.95–1.15) | 0.380 | 1.04 (0.95–1.14) | 0.401 |
| Sex | 1.15 (0.33–4.21) | 0.826 | 1.12 (0.34–3.85) | 0.852 |

Abbreviations: CI = confidence interval; CSVD = cerebral small vessel disease; MoCA = Montreal Cognitive Assessment; OR = odds ratio.

Supplementary Table 4. Full-process bootstrap sensitivity analysis of the preprocedural clinical plus CSVD burden model.

| **Metric** | **Apparent performance** | **Optimism** | **Optimism-corrected performance** |
| --- | --- | --- | --- |
| AUC | 0.890 | 0.080 | 0.810 |
| Brier score | 0.128 | -0.056 | 0.184 |
| Calibration slope | 1.000 | 0.482 | 0.518 |

This sensitivity analysis was performed using 1,000 bootstrap resamples. In each bootstrap sample, the entire model-building process was repeated, including 10-fold cross-validated LASSO variable selection at the lambda.min value, forced inclusion of age and sex, and refitting of the multivariable logistic regression model. Optimism was calculated as the mean difference in model performance between the bootstrap sample and the original dataset. For AUC and calibration slope, higher values indicate better performance; for Brier score, lower values indicate better prediction accuracy. This analysis was performed to account for uncertainty introduced by variable selection and should be interpreted as a more stringent sensitivity analysis than the fixed-model bootstrap validation reported in Table 3.

Abbreviations: AUC = area under the receiver operating characteristic curve; LASSO = least absolute shrinkage and selection operator.

Supplementary Table 5. Variable selection frequencies during bootstrap internal validation.

| **Variable** | **Selection frequency** |
| --- | --- |
| Age | Forced |
| Sex | Forced |
| Total CSVD burden score | 100.0% |
| Baseline MoCA score | 74.0% |
| Hypertension | 70.4% |
| Heart failure | 52.9% |
| HAS-BLED score | 51.6% |
| CHA₂DS₂-VASc score | 50.8% |
| LVDd | 46.8% |
| BMI | 45.7% |
| LAD | 44.9% |
| LVEF | 37.3% |
| PAF | 36.9% |
| DM | 36.8% |
| CAD | 36.6% |
| Prior stroke | 33.3% |

Selection frequencies were calculated across 1000 bootstrap resamples. In each bootstrap sample, 10-fold cross-validated LASSO variable selection was repeated at the lambda.min value. Age and sex were forced into all models a priori because of their clinical relevance and were therefore not considered LASSO-selected variables.

Abbreviations: BMI = body mass index; CAD = coronary artery disease; CSVD = cerebral small vessel disease; DM = diabetes mellitus; HF = heart failure; LAD = left atrial diameter; LASSO = least absolute shrinkage and selection operator; LVDd = left ventricular end-diastolic diameter; LVEF = left ventricular ejection fraction; MoCA = Montreal Cognitive Assessment; PAF = paroxysmal atrial fibrillation.

Supplementary Table 6. Mediation analysis of the association between CSVD burden score and 1-year MoCA-defined cognitive decline through perioperative SCE.

| **Effect** | **Type** | **Estimate** | **95% CI** | **P value** |
| --- | --- | --- | --- | --- |
| CSVD burden score → perioperative SCE | OR | 2.46 | 1.55–4.42 | <0.001 |
| Perioperative SCE →  cognitive decline | OR | 5.51 | 1.69–18.92 | 0.005 |
| Total effect | Effect size | 0.0786 | 0.0269–0.1100 | <0.001 |
| ACME | Effect size | 0.0137 | 0.0003–0.0300 | 0.040 |
| ADE | Effect size | 0.0649 | 0.0217–0.1000 | <0.001 |
| Proportion mediated | % | 17.4% | 0.4%–31.0% | 0.040 |

Abbreviations: ACME, average causal mediation effect; ADE, average direct effect; CI, confidence interval; CSVD, cerebral small vessel disease; MoCA, Montreal Cognitive Assessment; OR, odds ratio; SCE, silent cerebral embolism.

Perioperative SCE was defined as a new clinically silent diffusion-restricted ischemic lesion detected on early postprocedural MRI and was observed in 35 patients. ACME, ADE, total effect, and proportion mediated were estimated on the probability scale under the counterfactual mediation framework.

Supplementary Table 7. Mediation analysis of the association between total CSVD burden score and 1-year MMSE-defined cognitive decline through SCE.

| **Effect** | **Type** | **Estimate** | **95% CI** | **P value** |
| --- | --- | --- | --- | --- |
| CSVD burden score → SCE | OR | 3.47 | 2.08–5.81 | <0.001 |
| SCE → cognitive decline | OR | 3.67 | 1.08–12.52 | 0.038 |
| Total effect | Effect size | 0.0506 | 0.0134–0.0891 | <0.001 |
| ACME | Effect size | 0.0079 | -0.0004–0.0252 | 0.068 |
| ADE | Effect size | 0.0427 | 0.0118–0.0754 | <0.001 |
| Proportion mediated | % | 15.61% | -1.26%–37.17% | 0.068 |

MMSE-defined cognitive decline was defined as a decrease of ≥2 points in MMSE score from baseline to 12 months after LAAO. Mediation models were adjusted for age, sex, hypertension, and baseline MMSE score. Estimates for the total effect, ACME, and ADE represent absolute changes in the probability of cognitive decline.

Abbreviations: ACME = average causal mediation effect; ADE = average direct effect; CI = confidence interval; CSVD = cerebral small vessel disease; MMSE = Mini-Mental State Examination; SCE = silent cerebral embolism.

Supplementary Table 8. Model performance for predicting 1-year MMSE-defined cognitive decline.

| **Metric** | **Clinical model** | **Clinical + CSVD burden model** | **Clinical + CSVD burden model + SCE** |
| --- | --- | --- | --- |
| Apparent AUC | 0.671 | 0.886 | 0.900 |
| Optimism-corrected AUC | 0.609 | 0.856 | 0.866 |
| Apparent Brier score | 0.177 | 0.114 | 0.107 |
| Optimism-corrected calibration slope | 0.636 | 0.815 | 0.771 |
| DeLong P vs previous model | — | <0.001 | 0.264 |
| Likelihood ratio test P vs previous model | — | <0.001 | 0.035 |

Model performance was evaluated using MMSE-defined cognitive decline as the outcome. The clinical model included age, sex, hypertension, and baseline MMSE score. The clinical plus CSVD burden model additionally included total CSVD burden score, and the final model further included SCE. Internal validation was performed using 1,000 bootstrap resamples.

Abbreviations: AUC = area under the receiver-operating characteristic curve; CSVD = cerebral small vessel disease; MMSE = Mini-Mental State Examination; SCE = silent cerebral embolism.

Supplementary Figure 1. Decision curve analysis comparing the clinical model and the clinical plus CSVD burden model for predicting 1-year cognitive decline.

The clinical model included age, sex, hypertension, and baseline MoCA score. The clinical plus CSVD burden model additionally included the total CSVD burden score. Net benefit was plotted across threshold probabilities. The black horizontal line represents the treat-none strategy, and the gray line represents the treat-all strategy. The clinical plus CSVD burden model showed higher net benefit than the clinical model across most threshold probabilities, indicating improved clinical usefulness for individualized risk stratification.
